# Associations of dietary and lifestyle inflammation scores with sleep quality and mental health in hemodialysis patients: a multicenter cross-sectional study

**DOI:** 10.1186/s12986-025-00958-5

**Published:** 2025-07-01

**Authors:** Mohadeseh Soleimani Damaneh, Hossein Bavi Behbahani, Meysam Alipour, Ahmad Zare Javid, Sara Keramatzadeh, Shiva Shokri, Pardis Tofighzadeh, Fatemeh Fayazfar, Haleh Soltaniyan Dehkordi, Elahe Ghadimi, Siavash Babajafari Esfandabad, Shokouh Shayanpour

**Affiliations:** 1 Department of Nutrition, School of Public Health Iran University of Medical Sciences, Tehran, Iran; 2 Department of Nutrition, Shoushtar Faculty of Medical Sciences, Shoushtar, Iran; 3 Nutrition and Metabolic Diseases Research Center, Clinical Sciences Research Institute, Ahvaz Jundishapur University of Medical Sciences, Ahvaz, Iran; 4 Student Research Committee, Ahvaz Jundishapur University of Medical Sciences, Ahvaz, Iran; 5 Student Research Committee, School of Nutrition and Food Sciences, Shiraz University of Medical Science, Shiraz, Iran; 6 Student Research Committee, Shoushtar Faculty of Medical Sciences, Shoushtar, Iran; 7 Nutrition Research Center, School of Nutrition and Food Sciences, Shiraz University of Medical Science, Shiraz, Iran; 8 Department of Internal Medicine, Chronic Renal Failure Research Center, Ahvaz Jundishapur University of Medical Sciences, Ahvaz, Iran

**Keywords:** Renal dialysis, Sleep, Diet, Inflammatory, Mental health

## Abstract

**Background:**

Poor sleep quality and mental disorders are common issues among patients undergoing dialysis. Diet and lifestyle may be associated with sleep quality and mental health. The current study aimed to evaluate the associations between the Dietary and Lifestyle Inflammation Scale (DLIS) score and mental health and sleep quality among Iranian hemodialysis patients.

**Methods:**

This multicenter cross-sectional study was conducted on 423 patients undergoing hemodialysis at eight centers in three cities. The DLIS was calculated using information from a validated 168-item semiquantitative food frequency questionnaire. Mental health was evaluated via the 21-item Depression, Anxiety, and Stress Scale (DASS-21), and the Pittsburgh Sleep Quality Index (PSQI) was used to assess sleep quality. Other assessments included physical activity levels, biochemical parameters, and patient dialysis data. Statistical analyses via SPSS software were conducted to identify associations.

**Results:**

The mean ± standard deviation of age and body mass index (BMI) were 52.84 ± 14.63 years and 24.8 ± 5.11 kg/m^2^, respectively. A total of 58.9% of the participants were men. After controlling for potential confounders, participants in the top quartile of DLIS had greater odds of having poor sleep quality (OR: 3.18; 95% CI: 1.71–5.90), depression (OR: 1.94; 95% CI: 1.06–3.54), anxiety (OR: 2.82; 95% CI: 1.51–5.27), and stress (OR: 2.15; 95% CI: 1.14–4.03) than did those in the bottom quartile.

**Conclusion:**

Our findings revealed that increased dietary and lifestyle inflammatory potential, characterized by increased DLIS, was positively associated with increased risks of depression, anxiety, stress and poor sleep quality.

## Introduction

Chronic kidney disease (CKD) progresses to end-stage renal disease (ESRD), a severe condition that significantly contributes to global morbidity and mortality [1]. Hemodialysis (HD), the cornerstone treatment for ESRD, extends patient survival but introduces substantial challenges, including a high prevalence of sleep disorders and mental health conditions such as depression, anxiety, and stress-related disorders [2–5]. Over 80% of HD patients experience sleep disruptions, and the prevalence of depression is approximately 15% higher in dialysis patients than in kidney transplant recipients [6]. These disturbances reduce quality of life and exacerbate clinical outcomes by increasing the risk of cardiovascular disease, malnutrition, and systemic inflammation [7]. Despite the known burden of these comorbidities, uncertainties persist regarding the precise mechanisms linking ESRD, its treatment, and these adverse outcomes, particularly the role of modifiable factors such as diet and lifestyle.

Several factors, including age [8], sex [9], uremic pruritus [10], and dialysis duration [11, 12], influence sleep quality and mental health in HD patients. Among these factors, low-grade systemic inflammation has emerged as a critical mediator [13]. Biologically, inflammation disrupts neurotransmitter systems, increases oxidative stress, and dysregulates the hypothalamic‒pituitary‒adrenal (HPA) axis, all of which contribute to mood disorders and sleep disturbances [14, 15]. Elevated levels of proinflammatory cytokines, such as interleukin-6 (IL-6) and tumor necrosis factor-alpha (TNF-α), are associated with altered sleep architecture, reduced melatonin production, and increased psychological distress [16]. Furthermore, inflammation shifts tryptophan metabolism from serotonin production to the kynurenine pathway, promoting depressive symptoms [17]. While these mechanisms are well documented in healthy populations and individuals with other chronic diseases, their specific contributions in HD patients remain underexplored, highlighting a key knowledge gap.

Diet and lifestyle are pivotal in modulating systemic inflammation. The dietary inflammatory index (DII) links diet-related inflammation to depressive symptoms, anxiety, and poor sleep quality in various populations [18, 19]. However, the DII’s focus on individual nutrients overlooks broader dietary patterns and synergistic effects of food groups. To address this limitation, newer indices, such as the dietary inflammation score (DIS) and lifestyle inflammation score (LIS), incorporate dietary patterns, body mass index (BMI), physical activity, alcohol consumption, and smoking to provide a comprehensive assessment of inflammatory burden [20]. Higher DIS and LIS scores are associated with increased risks of chronic diseases such as cancer and diabetes [21–23], but their impact on mental health and sleep quality in HD patients has not been investigated. This gap is significant, as HD patients face unique inflammatory challenges due to dialysis and uremia, which may amplify the effects of proinflammatory diets and lifestyles.

The evidence from studies in healthy individuals and other patient populations underscores the importance of addressing inflammation to improve mental health and sleep outcomes. However, the applicability of these findings to HD patients is uncertain because of their distinct physiological and treatment-related inflammatory profiles. To the best of our knowledge, no study has explored the combined influence of dietary and lifestyle-related inflammation in this population. Thus, this study examined the associations between proinflammatory dietary and lifestyle patterns, quantified by the Dietary and Lifestyle Inflammation Scale (DLIS), and mental health disorders and sleep quality in HD patients. We hypothesize that higher DLIS scores are associated with greater psychological distress and poorer sleep quality. By elucidating these relationships, our research aims to inform targeted interventions to improve outcomes in this vulnerable population.

## Method

### Study population

This multicenter cross-sectional study included 423 HD patients from five governments and three private dialysis centers in Ahvaz, Shiraz, and Shushtar cities, Iran. The inclusion criteria included adult patients (aged ≥ 18 years) who received HD for at least 6 months. We excluded participants with enteral or parenteral feeding, active neoplastic disease, major amputation (lower/upper extremities), a diagnosis of cancer, acute or chronic pancreatitis, irritable bowel syndrome, prolonged gastrointestinal symptoms, acute or chronic pancreatitis, hepatic insufficiency, and energy intake less than 800 kcal/d or above 4200 kcal/d. The sample size for the study was determined via G*Power 3.1.9.4 software. On the basis of statistical parameters from a relevant previous study [24], including a significance level of 0.05, a power of 0.95, and an effect size of 0.33, the calculated minimum sample size was 389. To account for a 9% expected withdrawal rate, a total of 423 participants were enrolled in the study.

### Data collection

Sociodemographic and clinical data, including sex, age, marital status, time of dialysis start, frequency and duration of dialysis, fluid intake, urine volume, intradialytic weight gain, prescription medicines, and comorbidities (diabetes mellitus and hypertension), were extracted from the dialysis unit’s database. Diabetes was defined as a history of diabetes mellitus or the use of antidiabetic drugs. Hypertension was defined as the recording of hypertension in medical records or the use of antihypertensive drugs. Other factors, including BMI, job, education level, monthly income, physical activity, and smoking status, were gathered by researchers according to standard operating procedures or through a questionnaire administered via interviews. Digital scales were used to measure the participants’ body weight to the nearest 100 g with light clothes and without shoes at the end of the dialysis session. BMI was calculated from participants’ weight and height, which were measured at the end of their dialysis session.

A stadiometer was used to measure height to the nearest 0.5 cm standing while the individuals were barefoot. BMI was calculated as weight (kg) divided by height (m^2^). Physical activity was measured via the International Physical Activity Questionnaire (IPAQ), which contains seven questions, the validity of which has been confirmed in HD patients, and the results are expressed as metabolic equivalent hours per week (METs hr/wk) [25]. Biochemical indicators, including serum albumin, calcium, phosphate, creatinine, potassium, total iron-binding capacity, and urea nitrogen, were determined from the dialysis unit’s database for the same month. Dialysis adequacy was calculated on the basis of the Kt/V index via dialysis length, postdialysis weight, ultrafiltration volume, and pre- and postdialysis serum urea concentrations [26].

### Dietary assessment

Trained dietitians obtained information on the usual food intake of all participants via a valid and reliable 168-item semiquantitative food frequency questionnaire (FFQ) with standardized servings [27]. The participants were requested to provide information on the frequency of their consumption of each food item, indicating whether it was consumed daily, weekly, or monthly, over the preceding year. These reported frequencies were then converted into gram-weight equivalents. To determine the total energy and nutrient intake, Nutritionist IV software (Hearst Corporation) was utilized, with adjustments made to accommodate Iranian food items.

### Calculating the dietary and lifestyle inflammation score

The inflammatory scores of the participants were determined via dietary data derived from the FFQ. The DIS [28] is the sum of 19 weighted components, including 18 whole food and beverage groups and 1 micronutrient supplement score. DISs originally included leafy greens and cruciferous vegetables; tomatoes; apples and berries; deep yellow or orange vegetables; fruit; other fruits; natural fruit juices; other vegetables; legumes; fish; poultry; red and organ meats; processed meats; added sugars; high-fat dairy; low-fat dairy and tea; nuts; other fats; refined grains; starchy vegetables; and supplements. The mixed dishes were disaggregated into food groups. All of these components were used in addition to the supplement score because of the lack of information regarding supplement use among the study participants. To compute the DIS score, each food item was multiplied by its specific weight (explained in Byrd et al. study) to determine the weighted values of each item. The weighted values were then standardized via the Z score (to a mean of zero and an SD of 1). Finally, all the items’ standardized weighted values were summed to calculate the DIS score for the participants.

The LIS components include smoking status (“former/never” or “current”), physical activity (“high or moderate” and “low or no physical activity”), BMI (kg/m^2^) (“overweight (25–29.9)” and “obese (≥ 30)”), and alcohol intake. Alcohol intake was not included in the score because of the lack of information regarding the intake of alcohol in Iranian culture. Finally, all the weighted values were summed to calculate the LIS score. In this study, the DLIS for each subject was calculated by summing the DIS and LIS. A higher DLIS (more positive) indicates a more proinflammatory diet and lifestyle.

### Assessment of sleep quality and mental health

A Persian validated version of the Pittsburgh Sleep Quality Index (PSQI) was used to collect data on sleep hygiene [29]. The PSQI is a common tool for assessing sleep problems associated with anxiety, stress, depression, and schizophrenia. The selfreported questionnaire contains 19 questions divided into seven categories, including subjective sleep quality, sleep latency, sleep duration, habitual sleep efficiency, sleep disorders, the use of sleep medications, and daytime dysfunction caused by sleep disorders. The score of each part is 0–3, and the highest total score is 21. A global PSQI score over 5 is considered poor quality of sleep. The validity and reliability of this questionnaire have been investigated in Iran [30].

The Depression, Anxiety and Stress Scale (DASS-21) was used to screen for depression, anxiety and stress. The DASS-21 is a valid and reliable questionnaire for measuring the status of negative well-being in the general population. The questionnaire contains 21 items with 3 subscales. Each subscale includes 7 questions in which each item is rated on a 4-point Likert scale ranging from 0 to 3 to measure the severity of depression, anxiety and stress. The participants who achieved scores of ≥ 10, ≥8, and ≥ 15 were considered to have depression, anxiety, and stress, respectively [31].

### Statistical analysis

The statistical analysis was conducted via IBM SPSS Statistics software (Version 24) from IBM SPSS Statistics, Armonk, USA. The normality of variables was assessed via the Kolmogorov‒Smirnov test. The baseline characteristics of the individuals are expressed as the means ± SDs for continuous variables and percentages for categorical variables. The participants were also categorized according to quartiles of DLIS cutoff points. Differences in variables across DLIS quartiles were examined via one-way analysis of variance (ANOVA), and the chi-square test was used to compare categorical variables across quartiles. Energy and nutrient intakes by DLIS quartiles were evaluated via ANOVA.

Odds ratios (ORs) and 95% confidence intervals (CIs) were calculated to assess the risk of psychological disorders (depression, anxiety, stress) and poor sleep quality across quartiles of the DLIS, and multiple logistic regression was applied in crude and multivariable-adjusted models. In the first adjusted model, the confounding effects of center type, city, age, sex, diabetes status, hypertension status, job status, marital status, education, income status, interdialysis weight gain, dialysis vintage, dialysis time, frequency of hemodialysis sessions, fluid intake, and urine volume were controlled. Model 2 was additionally controlled for energy intake and medication prescriptions. A p value less than 0.05 was considered statistically significant.

## Results

**Fig. 1:**
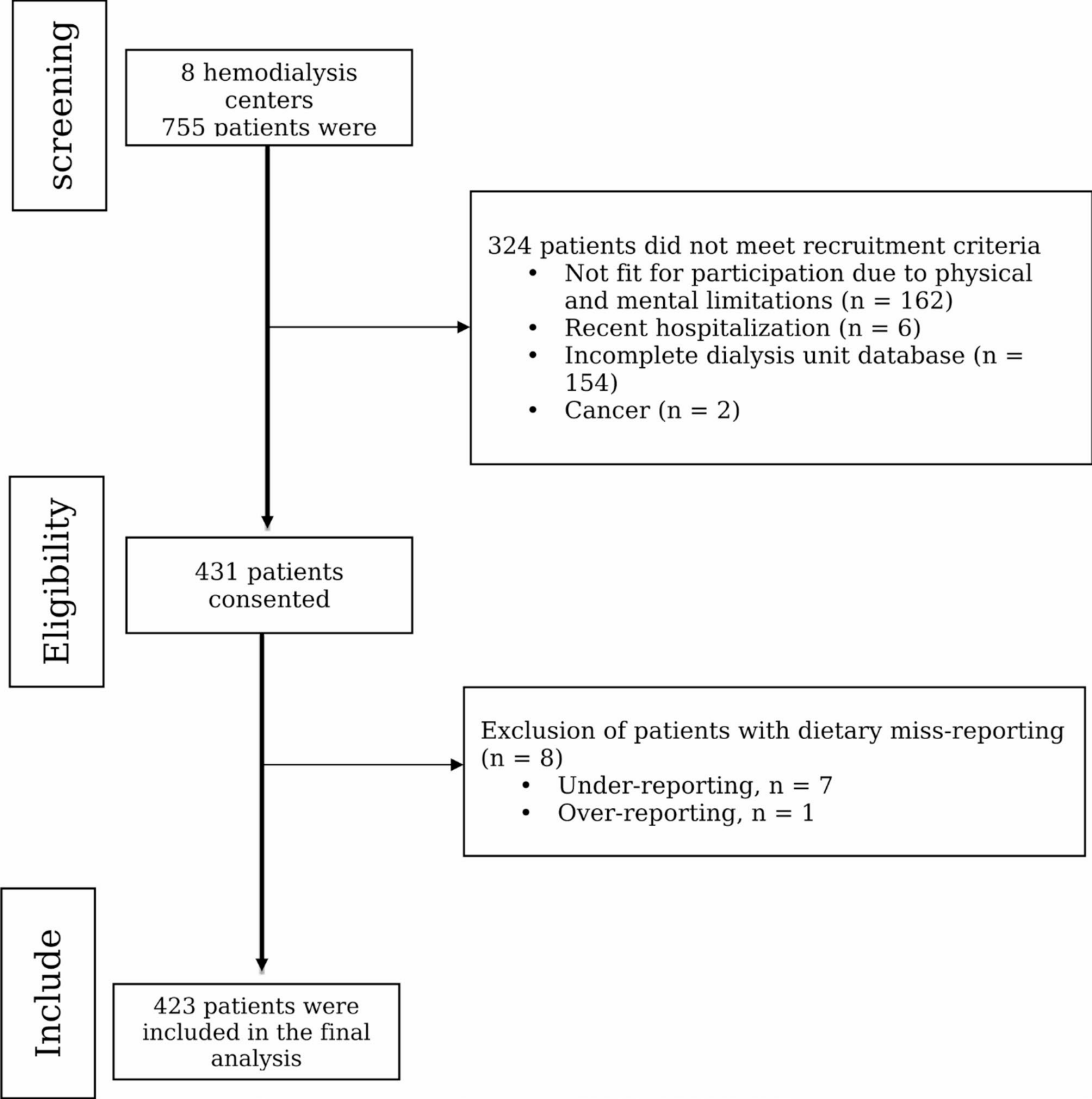
Participant flow chart

As shown in Fig. 1 and 324 of the 755 patients who were assessed across 8 hemodialysis centers were not included in the study for a variety of reasons. Thus, 431 patients in total consented to participate in the study. Owing to dietary misreporting, eight patients were excluded from the final study. Therefore, 423 patients were included in the final analysis. The mean ± SD age of the 423 HD patients included in the current study was 52.84 ± 14.63 years. Most patients were men (58.9%), married (73.8%), housekeepers (34.3%) or retired (23.2%). A total of 42.6% of the study participants had diabetes (*n* = 180), and 74.7% had hypertension (*n* = 316). The mean ± SD duration of dialysis was 49.41 ± 61.48 months. Moreover, the mean ± SD BMI was 24.8 ± 5.11 kg/m^2^. In terms of nutritional status, 8.4% of patients were underweight, 44.4% had a normal weight, 33.3% were overweight, and 13.4% were obese. A total of 10.2% of the patients in this study were smokers (*n* = 43). The prevalence of poor sleep quality in our study population was 60.5% (*n* = 256). Overall, 53.7% of the study participants were depressed (*n* = 227), 53.0% were anxious (*n* = 224), and 47.3% were stressed (*n* = 200).

Table 1 shows the general characteristics of the study population across quartiles of DLIS. The proportion of current smokers increased in the top quartile of the DLIS compared with the lowest quartile. Compared with those in the lowest quartile of the DLIS, significant differences were observed in the income status of the participants (*P* = 0.03). The participants in the highest quartile of the DLIS had significantly higher scores for all three mental health disorders (*P* < 0.001). Compared with participants in the first quartile of the DLIS, the number of participants with depression (*P* = 0.001), anxiety (*P* = 0.001), stress (*P* < 0.001), and poor sleep quality (*P* = 0.01) was greater in the highest quartile.

**Table 1:** Characteristics of patients undergoing hemodialysis across quartiles of dietary and lifestyle inflammation score (DLIS)

Characteristics	DLIS quartiles				*P* value
	Q1 (*N* = 108)	Q2 (*N* = 102)	Q3 (*N* = 103)	Q4 (*N* = 110)	
Score range	< – 0.51	-0.51- 0.64	0.65–1.75	1.75>	
Age, y	50.78 ± 14.76	54.68 ± 13.91	54.04 ± 15.19	52.05 ± 14.49	0.19
Male(%)	61(56.48)	56(54.9)	62(60.19)	69(62.73)	0.63
**Job**,** N(%)**					0.07
Unemployed	17(15.74)	11(10.78)	36(34.95)	23(20.91)	
Housekeeper	36(33.33)	40(39.22)	26(25.24)	33(30)	
Retired	23(21.3)	28(27.45)	2(1.94)	21(19.09)	
Employee	12(11.11)	2(1.96)	15(14.56)	12(10.91)	
Self-employment	18(16.67)	16(15.69)	7(6.8)	14(12.73)	
Others	2(1.85)	5(4.9)	36(34.95)	7(6.36)	
**City**					< 0.001
Ahvaz	44(40.74)	38(37.25)	42(40.78)	60(54.55)	
Shushtar	7(6.48)	15(14.71)	26(25.24)	21(19.09)	
Shiraz	57(52.78)	49(48.04)	35(33.98)	29(26.36)	
**Marital status**,** N(%)**					0.20
Married	71(65.74)	75(73.53)	76(73.79)	90(81.82)	
Single	29(26.85)	18(17.65)	19(18.45)	16(14.55)	
Divorced	6(5.56)	5(4.9)	3(2.91)	1(0.91)	
Dead spouse	2(1.85)	4(3.92)	5(4.85)	3(2.73)	
**Education**,** N(%)**					0.09
< 12 years	80(74.07)	78(76.47)	90(87.38)	86(78.18)	
≥ 12 years	28(25.93)	24(23.53)	13(12.62)	24(21.82)	
**Income status**,** N(%)**					0.03
< 5 million Rials	28(25.93)	32(31.37)	43(41.75)	31(28.18)	
5–10 million Rials	42(38.89)	47(46.08)	37(35.92)	42(38.18)	
10–20 million Rials	26(24.07)	19(18.63)	17(16.5)	33(30)	
> 20 million Rials	12(11.11)	4(3.92)	6(5.83)	4(3.64)	
Physical activity(MET/min/week)	864.13 ± 3388.29	417.87 ± 1189.7	183.47 ± 356.81	520.55 ± 2677.44	0.18
**Comorbidities**					
Diabetes(n, %)	40(37.04)	42(41.18)	49(47.57)	49(44.55)	0.44
Hypertension(n, %)	83(76.85)	76(74.51)	74(71.84)	83(75.45)	0.86
**Sleep**					
** Sleep duration**	6.63 ± 2.07	6.38 ± 2.94	6.33 ± 1.97	6.41 ± 3.03	0.83
** Sleep quality**					
** PSQI Score**	6.12 ± 3.93	6.61 ± 3.7	7.25 ± 3.56	7.18 ± 3.42	0.08
** Sleep quality category**					
good, N (%)	58(53.7)	43(42.16)	32(31.07)	34(30.91)	0.01
poor, N (%)	50(46.3)	59(57.84)	32(31.07)	76(69.09)	
**Depression**					
** Depression Score**	11.00 ± 11.07	10.39 ± 11.77	17.59 ± 14.19	14.56 ± 12.54	< 0.001
** Depression category**					
No, N (%)	57(52.8)	60(58.8)	35(34.0)	44(40.0)	0.001
Yes, N (%)	51(47.2)	42(41.2)	68(66.0)	66(60.0)	
**Anxiety**					
** Anxiety Score**	9.09 ± 9.11	9.51 ± 9.22	14.54 ± 14.22	13.98 ± 11.74	< 0.001
** Anxiety category**					
No, N (%)	64(59.3)	56(54.9)	38(36.9)	41(37.3)	0.001
Yes, N (%)	44(40.7)	46(45.1)	65(63.1)	69(62.7)	
**Stress**					
** Stress Score**	14.19 ± 12	13.35 ± 11.68	21.05 ± 12.42	17.75 ± 12.82	< 0.001
** Stress category**					
No, N (%)	64(59.3)	69(67.6)	39(37.9)	51(46.4)	< 0.001
Yes, N (%)	44(40.7)	33(32.4)	64(62.1)	59(53.6)	

Note: Data for quantitative variables are presented as means ± SD, obtained from ANOVA. Data for qualitative variables are presented as frequencies (percentages) and analyzed using chi-square tests

Abbreviations: DLIS, dietary and lifestyle inflammation score; PSQI, petersburg sleep quality questionnaire

The dialysis data of HD patients across quartiles of DLIS are presented in Table 2. The participants in the highest quartile of the DLIS had a higher frequency of dialysis per week and fluid intake. The other variables did not significantly differ across DLIS quartiles.

**Table 2:** Dialysis data of patients undergoing hemodialysis across quartiles of DLIS

Variables	DLIS quartiles				*P* value
	Q1 (*N* = 108)	Q2 (*N* = 102)	Q3 (*N* = 103)	Q4 (*N* = 110)	
Dialysis vintage, month	51.72 ± 56.5	52.02 ± 57.94	49.45 ± 81.72	44.72 ± 45.92	0.80
Dialysis time, hours	2.83 ± 0.49	2.88 ± 0.53	2.96 ± 0.6	2.85 ± 0.47	0.29
Frequency dialysis per week, Time/week	3.85 ± 0.48	3.74 ± 0.48	3.62 ± 0.57	3.57 ± 0.43	< 0.001
Fluid intake, ml	1370.64 ± 1457.42	1275.49 ± 996.86	976.7 ± 682.42	1251.45 ± 723.26	0.03
Urine volume, ml	429.91 ± 614.05	350.91 ± 523.02	299.26 ± 460.31	442.94 ± 556.55	0.18
Intradialytic weight gain, kg	2.05 ± 1.14	2.04 ± 1.19	1.93 ± 1.25	2.02 ± 1.17	0.90
**Laboratory parameters**					
Creatinine, mg/dL	8.31 ± 3.13	7.78 ± 4.49	7.54 ± 2.84	8.54 ± 4.34	0.19
Sodium, mmol/L	139.82 ± 3.81	138.83 ± 5.06	138.91 ± 4.27	140.12 ± 6.24	0.14
Potassium, mmol/L	5.18 ± 0.87	5.04 ± 0.86	4.99 ± 0.84	5 ± 0.74	0.33
Calcium, mg/dL	8.38 ± 0.96	8.33 ± 1	8.55 ± 0.72	8.34 ± 0.89	0.65
Phosphate, mg/dL	5.27 ± 1.51	5.07 ± 1.38	4.97 ± 1.22	5.35 ± 1.34	0.15
Kt/V	1.28 ± 0.52	1.25 ± 0.63	1.21 ± 0.41	1.17 ± 0.46	0.37
Albumin, g/ dL	5.92 ± 19.15	4.08 ± 0.57	4.1 ± 0.76	4.17 ± 0.6	0.42
Serum total iron binding capacity, µg/dL	283.51 ± 90.26	294.45 ± 104.36	305.63 ± 85.59	315.64 ± 96.55	0.07
**Medication prescriptions**					
Calcium carbonate 500 mg, time/day	1.59 ± 1.93	1.18 ± 1.71	1.13 ± 1.81	1.34 ± 1.8	0.24
Sevelamer hydrochloride 800 mg, time/day	0.81 ± 1.47	1.1 ± 1.67	0.73 ± 1.27	0.83 ± 1.5	0.29
Calcitriol 0.25 mcg, time/day	0.92 ± 1.38	0.52 ± 0.89	0.7 ± 1.17	0.58 ± 1.14	0.06
furosemide time/day	0.38 ± 0.86	0.51 ± 1.13	0.43 ± 0.95	0.32 ± 0.8	0.47
Corticosteroids, N(%)	8(7.41)	2(1.96)	3(2.91)	3(2.73)	0.14
Lipid-lowering drugs, N(%)	21(19.44)	16(15.69)	18(17.48)	13(11.82)	0.46

Note: The data are presented as “mean ± SD”

The significant difference based on one-way ANOVA (*P* < 0.05)

The dietary intakes and DIS and LIS components of the participants according to quartiles of DLIS are presented in Table 3. Compared with participants in the lowest quartile, those in the highest quartile of the DLIS had significantly greater intakes of energy and protein (*P* = 0.002), while the intakes of carbohydrates (*P* < 0.001) and fat (*P* = 0.006) were lower. Additionally, BMI increased significantly across DLIS quartiles (*P* < 0.001).

**Table 3:** Dietary intakes, DIS and LIS components of patients undergoing hemodialysis according to quartiles of the DLIS

Variables	DLIS quartiles				*P* value
	Q1 (*N* = 108)	Q2 (*N* = 102)	Q3 (*N* = 103)	Q4 (*N* = 110)	
**Nutrient Intake**					
Energy(Kcal/d)	2391.66 ± 1002.83	2112.34 ± 724.5	2002.61 ± 662.67	2422.35 ± 896.42	< 0.001
Carbohydrate(g/d)	89.48 ± 43.28	77.84 ± 32.12	71.82 ± 26.99	80.77 ± 31.3	0.002
Protein(g/d)	349.4 ± 149.92	309.61 ± 107.22	302.59 ± 114.47	375.79 ± 153.98	< 0.001
Total fat(g/d)	75.34 ± 44.09	64.87 ± 31.79	58.09 ± 25.94	68.25 ± 38.02	0.006
**DIS component**					
Leafy greens and cruciferous vegetables(g/d)	35.93 ± 58.28	29.43 ± 45.01	25.22 ± 19.87	34.97 ± 30.83	0.19
Tomatoes(g/d)	28.89 ± 38.67	15.6 ± 22.28	16.87 ± 20.49	20.53 ± 27.17	0.003
Apples and berries(g/d)	46.42 ± 42.02	42.34 ± 38.92	49.75 ± 93.88	36.71 ± 32.24	0.37
Deep yellow or orange vegetables and fruit(g/d)	47.4 ± 61.01	42.16 ± 66.95	51.74 ± 75.09	77.09 ± 114.09	0.01
Other fruits and real fruit juices(g/d)	163.9 ± 161.83	175.78 ± 157.92	174.52 ± 138.04	189.34 ± 212.22	0.74
Other vegetables(g/d)	80.68 ± 88.9	64.5 ± 55.92	65.57 ± 55.51	68.7 ± 52.6	0.24
Legumes(g/d)	56.84 ± 43.15	56.51 ± 38.86	54.33 ± 42.74	59.51 ± 48.48	0.85
Fish(g/d)	13.68 ± 17.46	12.76 ± 15.29	14.32 ± 21.74	22.91 ± 31.75	0.003
Poultry(g/d)	49.53 ± 65.36	42.55 ± 45.7	49.95 ± 70.95	55.49 ± 61.9	0.50
Red and organ meats(g/d)	18.48 ± 23.18	15.25 ± 13.77	13.87 ± 12.08	20.26 ± 26.98	0.08
Processed meats(g/d)	4.94 ± 10.75	4.1 ± 6.92	3.94 ± 8.09	4.97 ± 8.81	0.75
Added sugars(g/d)	71.6 ± 125.83	61.68 ± 77.52	86.83 ± 110.33	76.19 ± 83.4	0.35
High-fat dairy(g/d)	61.59 ± 75.69	59.01 ± 87.59	64.79 ± 84.71	67.61 ± 86.81	0.88
Low-fat dairy(g/d)	107.35 ± 169.36	89.57 ± 126.87	100 ± 110.5	95.38 ± 150.23	0.82
Coffee and tea(g/d)	346.32 ± 304.14	344 ± 312.76	338.14 ± 346.14	354.29 ± 301.24	0.98
Nuts(g/d)	6.58 ± 7.55	6.85 ± 8.23	8.17 ± 13.8	12.98 ± 31.76	0.03
Other fats(g/d)	29.28 ± 22.15	23.4 ± 17.44	26.44 ± 18.69	29.86 ± 23.83	0.09
Refined grains and starchy vegetables(g/d)	457.31 ± 212.62	464.27 ± 267.68	448.04 ± 291.83	478.6 ± 267.69	0.85
**LIS component**					
BMI(kg/m^2^)	24.34 ± 4.35	23.21 ± 3.95	24.86 ± 4.58	26.82 ± 6.47	< 0.001
Underweight	8(7.41)	16(15.69)	7(6.8)	6(5.45)	< 0.001
Normal weight	60(55.56)	56(54.9)	49(47.57)	23(20.91)	
Overweight	29(26.85)	29(28.43)	32(31.07)	51(46.36)	
Obese	11(10.19)	1(0.98)	15(14.56)	30(27.27)	
Physical activity; (MET-h/d)					0.68
low	91(84.26)	81(79.41)	86(83.5)	92(83.64)	
moderate	17(15.74)	19(18.63)	14(13.59)	16(14.55)	
Sever	91(84.26)	2(1.96)	3(2.91)	2(1.82)	
Current smoker; n(%)	8(7.41)	6(5.88)	91(88.35)	17(15.45)	0.08

Note: Data for quantitative variables are presented as means ± SD, obtained from ANOVA. Data for qualitative variables are presented as frequencies (percentages) and analyzed using chi-square tests

Abbreviations: DIS, dietary inflammation score; DLIS, dietary and lifestyle inflammation score; LIS, lifestyle inflammation score; BMI, body mass index

The associations of the DLIS score with the risk of poor sleep quality, depression, anxiety, and stress are shown in the three models in Table 4. In the first model (univariate model), the odds of poor sleep quality were greater in individuals in the highest quartiles of the DLIS (OR: 2.59; 95% CI: 1.49, 4.51; P value < 0.001). This significant positive association between DLIS and poor sleep quality persisted in the fully adjusted model that controlled for city, age, sex, diabetes, hypertension, job, marital status, education, income status, interdialysis weight gain, dialysis vintage, dialysis time, frequency of hemodialysis sessions, fluid intake, urine volume, energy intake, and medication prescriptions (OR: 3.18, 95% CI: 1.71, 5.9; P value < 0.001). In addition, in the crude model, compared with those in the bottom quartile, individuals in the top quartile of the DLIS had a significantly greater risk of depression (OR: 1.68; 95% CI: 0.98, 2.87; P value = 0.004), anxiety (OR: 2.45; 95% CI: 1.42, 4.22; P value < 0.001), and stress (OR: 1.68; 95% CI: 0.98, 2.88; P-trend = 0.002). A similar pattern was shown in the third model (full model multivariate analysis) for depression, anxiety, and stress. There was a significant positive association between DLIS and increased likelihood of depression (OR: 1.94; 95% CI: 1.06–3.54), anxiety (OR: 2.82; 95% CI: 1.51–5.27), and psychological distress (OR: 2.15; 95% CI: 1.14–4.03).

**Table 4:** Crude and multivariable-adjusted odds ratio (95% CI) of the associations between DLIS and sleep quality, stress, anxiety and depression

	DLIS quartiles				
	Q1(*N* = 108)	Q2(*N* = 102)	Q3(*N* = 103)	Q4(*N* = 110)	*P*-value
**Sleep quality**					
Model 0^*a*^	1(ref)	1.59(0.92, 2.75)	2.57(1.47, 4.52)	2.59(1.49, 4.51)	< 0.001
Model 1^*b*^	1(ref)	1.52(0.85, 2.7)	2.62(1.42, 4.82)	2.97(1.62, 5.43)	< 0.001
Model 2^*c*^	1(ref)	1.64(0.9, 3)	2.79(1.49, 5.25)	3.18(1.71, 5.90)	< 0.001
**Depression**					
Model 0^*a*^	1(ref)	0.78(0.45, 1.35)	2.17(1.25, 3.79)	1.68(0.98, 2.87)	0.004
Model 1^*b*^	1(ref)	0.68(0.38, 1.22)	2.03(1.11, 3.73)	1.92(1.07, 3.47)	0.002
Model 2^*c*^	1(ref)	0.74(0.4, 1.35)	2.06(1.11, 3.83)	1.94(1.06, 3.54)	0.003
**Anxiety**					
Model 0^*a*^	1(ref)	1.19(0.69, 2.07)	2.49(1.43, 4.33)	2.45(1.42, 4.22)	< 0.001
Model 1^*b*^	1(ref)	1.12(0.62, 2.03)	2.47(1.34, 4.55)	2.74(1.49, 5.03)	< 0.001
Model 2^*c*^	1(ref)	1.15(0.62, 2.13)	2.45(1.32, 4.57)	2.82(1.51, 5.27)	< 0.001
**Stress**					
Model 0^*a*^	1(ref)	0.70(0.40, 1.22)	2.39(1.37, 4.15)	1.68(0.98, 2.88)	0.002
Model 1^*b*^	1(ref)	0.63(0.34, 1.18)	2.59(1.39, 4.83)	2.06(1.12, 3.79)	0.001
Model 2^*c*^	1(ref)	0.68(0.36, 1.29)	2.54(1.33, 4.84)	2.15(1.14, 4.03)	0.001

DLIS: dietary and lifestyle inflammation score

*P* < 0.05 statistically significant by multivariable logistic regression

Model 0. binary logistic regression analysis without adjustment

Model 1. binary logistic regression analysis with center type, city, age, sex, diabetes, hypertension, job, marital status, education, income status, inter-dialysis weight gain, dialysis vintage, dialysis time, frequency of hemodialysis sessions, fluid intake, and urine volume

Model 2. binary logistic regression analysis with adjustment for model 2 in addition to energy intake, and medication prescriptions (corticosteroids, lipid-lowering drugs, calcium carbonate, calcitriol, sevelamer hydrochloride, frusemide)

## Discussion

In this multicenter cross-sectional study, we investigated the associations between adherence to a proinflammatory diet and lifestyle, as measured by the Dietary and Lifestyle Inflammation Scale (DLIS), mental health disorders (stress, anxiety, depression) and sleep quality among Iranian maintenance hemodialysis patients. Our findings indicate a significant association between higher DLIS scores and poorer sleep quality and an increased prevalence of mental health disorders, which is consistent with the emerging global literature on the role of inflammation in these outcomes.

Our study aligns with recent research highlighting the link between proinflammatory diets and adverse health outcomes in chronic disease populations. For example, a meta-analysis by Wang et al. involving 92,242 participants across Asia, Europe, America, and Australia revealed that higher dietary inflammatory index (DII) scores were associated with increased depression, anxiety, and distress [32]. Similarly, a cross-sectional analysis of data from 4,232 CKD patients from the 2007–2018 NHANES dataset revealed that pro-inflammatory diets, assessed via the Dietary Inflammatory Index (DII), were significantly associated with increased depression symptoms. The association remained particularly strong in older adults, males, smokers, and individuals without sleep disorders, suggesting that dietary inflammation may play a key role in mental health among CKD patients [33]. Unlike these studies, which primarily used the DII, our study employed the DLIS, which integrates both dietary and lifestyle factors (e.g., smoking, BMI, and physical activity), offering a more holistic assessment of inflammation drivers. In contrast, a cross-sectional study by Rostami et al. in 400 Iranian health professionals revealed no association between the anti-inflammatory MIND diet and mental health outcomes, potentially because of its focus on a healthier population and reliance on self-reported data, which may introduce recall bias [34]. Our study focused on HD patients, a population with unique dietary restrictions and high inflammation, distinguishing them from these works, as does the use of DLIS, which captures lifestyle factors not addressed by DII or MIND.

Compared with a study by Jansen et al. in 4,467 Mexican women, which linked a proinflammatory modern Mexican diet (high in tortillas and soda and low in fiber) to a 23% greater likelihood of poor sleep quality [35], our findings extend this association to HD patients, where dietary restrictions exacerbate unhealthy eating patterns. However, unlike Jansen et al., we adjusted for confounders such as comorbidities and physical activity, strengthening causal inference. In contrast, a cross-sectional study from Isfahan, Iran, involving 160 hemodialysis patients reported that although 84% experienced poor sleep quality, there was no significant association between the intake of macro- and micronutrients—including vegetables—and sleep quality, highlighting the need for more comprehensive lifestyle assessments [24]. Our study’s novelty lies in its application of DLIS to HD patients, a relatively understudied group in the context of combined dietary and lifestyle inflammation indices.

The DLIS offers advantages over other indices, such as DII, DIS, and LIS, by combining dietary and lifestyle components. Previous studies, such as an analysis by Byrd et al., demonstrated that DLIS had a stronger correlation with circulating inflammatory markers (e.g., CRP and IL-6) than did DII in three U.S. populations, likely due to its inclusion of lifestyle factors such as smoking and BMI [36]. While the DII focuses on nutrient-specific inflammation and DIS on dietary components, it overlooks nondietary contributors, which the LIS addresses without dietary integration. Furthermore, a cross-sectional study conducted in Tehran, Iran, revealed that higher DLIS scores were significantly associated with increased odds of metabolic syndrome (MetS). Compared with those in the lowest quartile, participants in the highest DLIS quartile had a 57% greater likelihood of MetS, even after adjusting for potential confounders [37]. However, direct comparisons of DLIS’s predictive power for sleep and mental health outcomes in HD patients are lacking. We recommend further validation studies to compare DLIS with DII, DIS, and LIS in HD populations to confirm its applicability and superiority in this context.

The associations between pro-inflammatory diets and lifestyles (higher DLIS scores) and poor sleep quality and mental health disorders in HD patients are mediated by complex, interrelated biological pathways. Proinflammatory diets high in processed foods and low in fiber, combined with lifestyle factors such as smoking, high BMI, and low physical activity, increase systemic inflammation by increasing the levels of proinflammatory cytokines (e.g., TNF-α, IL-6, and CRP) and adipokines, which disrupt sleep architecture by altering sleep regulating peptides such as orexin and activating the hypothalamic‒pituitary‒adrenal (HPA) axis, leading to sleep fragmentation and reduced sleep efficiency [14–16]. These cytokines also contribute to neuroinflammation, impairing the serotonin and dopamine pathways critical for mood regulation, thus increasing depression and anxiety risk [38]. In HD patients, uremic toxins and dialysis-related stress amplify these inflammatory effects [39]. Neuroendocrine pathways are also affected, with pro-inflammatory diets and smoking disrupting circadian rhythms by reducing melatonin production, impairing sleep onset, and increasing sympathetic nervous system activity, which elevates catecholamines that heighten anxiety and disrupt sleep [40, 41]. HPA axis dysregulation, which is common in HD patients, leads to hypercortisolemia, a risk factor for depression [42]. Lifestyle factors further exacerbate these outcomes: increased adipose tissue from high BMIs, which is prevalent in HD patients with limited mobility, produces proinflammatory adipokines linked to poor sleep and mental health issues, although some studies have reported no link between obesity and diet-induced inflammation (e.g., via DII) due to population differences or unmeasured confounders such as sociocultural factors [43, 44]. Low physical activity, typical in HD patients, reduces the release of anti-inflammatory cytokines (e.g., IL-10) and endorphin, which promote relaxation and sleep; however, a 2022 systematic review confirmed that exercise improves sleep by regulating circadian rhythms and reducing stress [45, 46]. Smoking elevates CRP and oxidative stress, disrupting metabolism and contributing to sleep disturbances and mental health issues [47, 48]. A bidirectional relationship exists between inflammation and mental health, where depression and anxiety promote proinflammatory behaviors (e.g., unhealthy eating), and inflammation exacerbates mental health issues via neuroinflammatory pathways [49]. A 2021 meta-analysis by Firth et al. revealed that smoking was causally linked to mental disorders, whereas physical activity was protective [50]. Obesity was identified as a mental health risk factor in a 2023 Austrian registry study [51]. The prevalence of mental health disorders among HD patients is notably high. A study reported that 63.9% of HD patients experienced anxiety, 60.5% had depression, and 51.7% reported stress [52]. Discrepancies with studies such as Rostami et al. [34], which reported no link between the MIND diet and mental health, may reflect healthier populations, male-only samples, or recall bias from self-reported data. Our study’s use of DLIS and focus on HD patients mitigate some issues, but it is important to interpret our findings with caution due to potential unmeasured confounders (e.g., sleep apnea and financial stress). These factors may have contributed to the observed associations and should be addressed in future research.

### Strengths and limitations

This study has several limitations that should be acknowledged. First, although the sample size is comparable to those used in similar cross-sectional studies, a larger cohort would have provided greater statistical power and increased the robustness of the findings. Second, the study population consisted of individuals undergoing hemodialysis within a limited geographic and clinical context, which may restrict the generalizability of the results to broader or more diverse populations. Third, dietary intake was assessed via a single method—the FFQ. While this tool is widely used and validated for estimating habitual intake, reliance on self-reported data introduces the potential for recall bias and measurement error. Additionally, although we adjusted for key confounders, we did not account for certain factors, including financial stress, social support, antidepressants, sleep apnea, restless leg syndrome, and dialysis burden, which may influence inflammation, sleep quality and mental health outcomes. Furthermore, the DLISs were calculated via U.S.-derived weights, which may not fully capture dietary or lifestyle patterns relevant to Iranian populations. Moreover, sex-specific analyses were not conducted, which limits the exploration of potential sex-related differences in the associations. Finally, the cross-sectional design of the study precludes causal inference, limiting the ability to determine the directionality of the relationships observed between diet, lifestyle, sleep quality, and mental health outcomes.

Despite these limitations, this study provides meaningful insights into the role of an inflammatory diet and lifestyle patterns in sleep and mental health among patients undergoing hemodialysis and highlights important areas for further investigation.

### Future directions

Future research should focus on longitudinal studies to establish causality between DLIS and mental health outcomes in hemodialysis patients. Randomized controlled trials (RCTs) are needed to evaluate whether anti-inflammatory diets and lifestyle interventions can improve sleep quality and psychological well-being in this population. Additionally, incorporating inflammatory biomarkers in future studies would help clarify the biological mechanisms linking inflammation to mental health. Further validation of the DLIS in diverse populations, particularly in non-Western dietary contexts, is essential to enhance its clinical applicability. These efforts will help determine whether dietary and lifestyle modifications should be integrated into routine nephrology care for better patient outcomes.

## Conclusion

This study revealed a significant association between higher dietary and lifestyle inflammation scores (DLISs) and increased risks of poor sleep quality, depression, anxiety, and stress in hemodialysis patients. These findings suggest that proinflammatory diets and lifestyles may be important, modifiable factors for improving mental health and sleep outcomes in HD patients. While causality cannot be established due to the cross-sectional nature of the study, these results provide a strong foundation for future interventional research. If validated in longitudinal and clinical trials, dietary and lifestyle modifications could be integrated into nephrology guidelines and public health policies to increase patient care and quality of life.

## Data Availability

No datasets were generated or analysed during the current study.
